# Epimedium-Derived Multi-Antioxidant Carbon Dots Nanozymes for Mitigating Drought Stress of Ginseng Seedlings

**DOI:** 10.3390/plants14233705

**Published:** 2025-12-04

**Authors:** Yanghong Liu, Tong Wu, Jialong He, Chunyao Shang, Jiaheng Li, Yu Dong, Huiyuan Xie, Chen Xu, Yingping Wang, Kai Dong

**Affiliations:** 1 College of Chinese Medicinal Materials, Jilin Agricultural University, Changchun 130118, China; 2 National & Local Joint Engineering Research Center for Ginseng Breeding and Development, Changchun 130118, China

**Keywords:** carbon dots, nanozyme, ginseng, drought stress, reactive oxygen species

## Abstract

Drought stress induces oxidative damage that severely impairs the growth and development of ginseng seedlings. Although conventional antioxidants present a theoretical approach for mitigating such oxidative damage, their practical application is constrained by their inadequate stability. Herein, we developed multifunctional antioxidant carbon dots (CDs) synthesized from the medicinal herb *Epimedium* via a one-step hydrothermal method. The biomass-derived CDs exhibited efficient cascade nanozyme activities for mimicking both superoxide dismutase and catalase to achieve effective scavenging of multiple reactive oxygen species (ROS). Under drought stress, application of CDs to ginseng seedlings significantly mitigated oxidative damage through the modulation of the antioxidant enzyme system and improved osmotic regulation. Simultaneously, it could enhance photosynthetic efficiency and mitigate growth suppression caused by drought. Transcriptomic analysis revealed that CDs alleviated drought stress by triggering transcriptional reprogramming that activated genes related to antioxidant defense, photosynthetic efficiency, and stress signaling. Additionally, the CDs exhibited excellent biocompatibility and environmental safety. This work provides a novel and environmentally friendly strategy to enhance drought tolerance in medicinal plants.

## 1. Introduction

Global climate change is worsening droughts through rising temperatures and reduced rainfall [1]. Simultaneously, outdated and inefficient irrigation systems in many regions are further aggravating water shortages [2]. It is estimated that by 2030, at least 20% of developing countries will experience severe water scarcity, posing a substantial threat to global food security and ecosystem stability [3]. Drought not only endangers food production but also severely impacts traditional Chinese medicinal herbs, which often require specific ecological conditions to thrive [4,5]. Among them, the perennial medicinal herb ginseng (*Panax ginseng* C.A. Meyer) is particularly vulnerable [6]. The seedling stage is especially critical, as it establishes the foundation for the entire growth cycle [7]. This phase influences root development, shoot morphology, and subsequent nutrient uptake and utilization, ultimately determining the final yield and quality of ginseng. Therefore, enhancing plant resilience to drought is crucial for promoting green agricultural development and ensuring the stability of medicinal plant production.

Under drought stress, plant cellular homeostasis is significantly perturbed [8]. The reactive oxygen species (ROS), such as the superoxide anion (O_2_^•−^), hydrogen peroxide (H_2_O_2_), singlet oxygen (^1^O_2_), and the highly reactive hydroxyl radical (·OH), are excessively generated [9]. The excessive accumulation of ROS triggers a cascade of damage to cellular components, including membrane lipids, proteins, and nucleic acids, resulting in severe harm to plants and potentially leading to plant mortality [10]. To mitigate oxidative stress-triggered damage, antioxidants such as vitamins, melatonin, polyphenols, carotenoids, and anthocyanins are commonly employed to scavenge ROS. Nevertheless, the efficacy of such antioxidants remains constrained by their inherent instability and susceptibility to degradation under prevalent environmental factors including illumination, thermal stress, and humidity [11]. Consequently, it is essential to develop innovative alternatives to address these challenges associated with conventional antioxidants.

The application of nanotechnology has recently shown great potential in improving plant stress tolerance [12]. Among various nanomaterials, nanozymes are defined by their unique ability to mimic enzymatic functions [13]. Crucially, nanozymes offer advantages such as stable catalytic activity under extreme conditions, and excellent storage and transport stability, positioning them as superior alternatives to natural enzymes. Metal nanoenzymes (such as ZnO and Fe_3_O_4_ [14]) have demonstrated efficacy in scavenging ROS and promoting plant growth. However, their synthesis generally relies on complex, multi-step processes that necessitate organic solvents or harsh chemical reagents, making these methods often incompatible with the principles of green chemistry. Moreover, as single-atom nanozymes [15], they typically possess high specificity toward a particular ROS, which renders them inadequate for countering the diverse spectrum of ROS generated during cellular oxidative stress and thus limits their effectiveness in mitigating comprehensive oxidative damage. Consequently, the development of novel, green, multifunctional biomimetic antioxidant composites is imperative.

Carbon dots (CDs), as an emerging class of carbon-based nanozymes, exhibit substantial application potential in the fields of biomedicine and agriculture owing to their small size, tunable optical properties and excellent biocompatibility [16,17]. In particular, biomass-derived CDs are attracting great interest because they offer an eco-friendly solution [18]. Previous studies have confirmed that CDs synthesised from biomass sources such as fruit peels inherently possess enzyme-mimetic activity [19]. As natural reservoirs of antioxidant constituents, traditional Chinese medicinal herbs demonstrate even more distinctive potential for preparing CDs with augmented antioxidant activity [20]. Epimedium as a classic medicinal herb is abundant in flavonoids and phenolic acid compounds possessing potent antioxidant activity [21], rendering it an ideal precursor for synthesising antioxidant CDs. Based on this principle, we hypothesise that CDs derived from epimedium possess broad-spectrum ROS scavenging capabilities. Not only can they mimic multi-enzyme activity (specifically superoxide dismutase and catalase, i.e., SOD and CAT), but they can also effectively eliminate other free radicals.

Herein, we synthesized multifunctional antioxidant CDs through a simple one-step hydrothermal method using *Epimedium* as the carbon source. The CDs demonstrated significant cascade nanozyme activities mimicking both SOD and CAT, as well as potent scavenging abilities against multiple free radicals such as ABTS^+^· and ·OH. The efficient ROS scavenging capacity of the CDs was demonstrated in vitro and subsequently substantiated in ginseng plants under drought stress. Pot experiments demonstrated that CDs eliminated ROS by modulating antioxidant enzymes and enhancing osmotic regulation in ginseng seedlings. Consequently, it significantly enhanced photosynthetic efficiency, thereby alleviating the drought-induced growth inhibition. Furthermore, transcriptomic analysis revealed that CDs mitigated drought stress by modulating a complex network of genes responsible for enhancing antioxidant defense, photosynthetic efficiency, and stress signaling pathways. The CDs also showed excellent biocompatibility and environmental safety. In conclusion, *Epimedium*-derived CDs significantly enhanced the drought tolerance of ginseng seedlings, offering a promising strategy to enhance resilience in medicinal plants.

## 2. Results

### 2.1. Preparation and Characterization of Epimedium-Derived CDs

**Figure 1:**
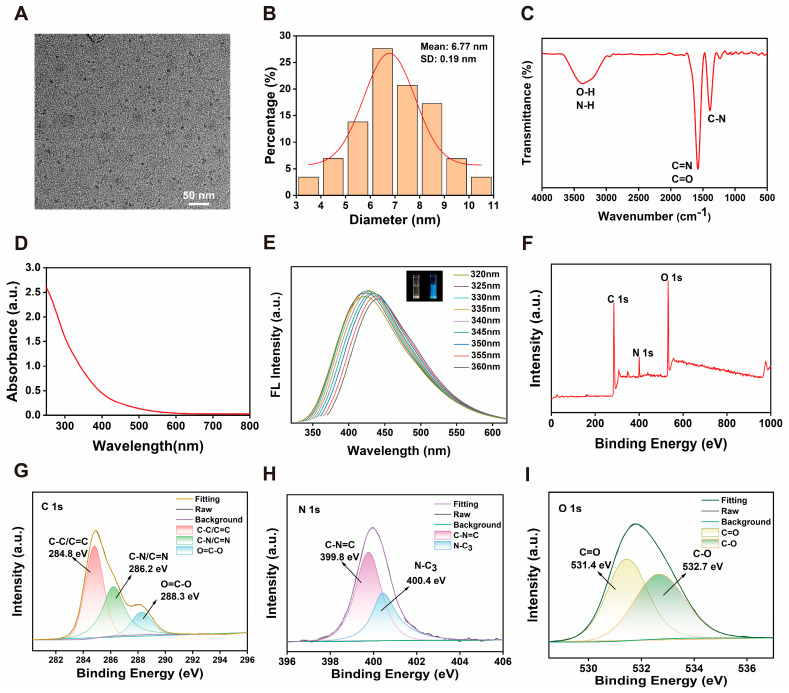
(**A**) TEM image of CDs, scale bar = 50 nm. (**B**) Size distribution of CDs. (**C**) FTIR spectra of CDs. (**D**) UV-vis absorption spectra. (**E**) Fluorescence emission spectra of CDs solution at different excitation wavelengths (320–360 nm) (Inset: CDs solution under daylight and 365 nm UV light). (**F**) XPS survey spectrum of CDs and (**G**–**I**) high-resolution C 1s, N 1s, and O 1s spectra.

As shown in Figure 1A, the morphology of the *Epimedium*-derived CDs was characterized by transmission electron microscopy (TEM). The images revealed that the CDs exhibited good dispersion, with all particles measuring less than 10 nm in size. Consistently, the particle size distribution profile of the CDs was presented in Figure 1B and the average diameter of CDs was 6.77 ± 0.19 nm. To identify the surface functional groups of the CDs, Fourier transform infrared (FTIR) spectroscopy was performed (Figure 1C). Notably, the FTIR spectrum exhibited a broad absorption band at 3370 cm^−1^, attributed to the stretching vibrations of O-H and N-H groups. Meanwhile, the peaks at 1576 cm^−1^ and 1387 cm^−1^ were assigned to the stretching vibrations of C=N/C=O and C-N bonds, respectively. The presence of these functional groups suggested that the CDs surface was hydrophilic, which is advantageous for biological applications. Additionally, the optical properties of the CDs were investigated by UV-visible absorption and fluorescence spectroscopy (Figure 1D,E). Figure 1D exhibited a broad absorption peak between 200 and 300 nm, which corresponded to the π–π* transition of the aromatic C=C bonds and indicated the formation of a graphitic carbon core in the CDs. With the ultraviolet light irradiation, the CD solution displayed strong blue fluorescent emission. The fluorescence spectrum of CDs showed a maximum emission peak at 430 nm under 335 nm excitation. Moreover, the CDs exhibited excitation-dependent emission behavior when excited from 320 to 360 nm. As depicted in Figure 1E, the emission maximum underwent a progressive red-shift accompanied by increasing excitation wavelength, which is indicative of multiple emissive centers distributed on the surface of CDs. In addition, the CDs solution retained its fluorescence intensity after 60 days (Figure S1), demonstrating good stability and the potential for long-term storage.

Furthermore, XPS analysis was used to confirm the structure and presence of the constituent elements and the chemical composition of the CDs. Figure 1F revealed three characteristic peaks for C 1s, O 1s and N 1s. Specifically, deconvolution analysis of the C 1s XPS spectrum (Figure 1G) revealed six distinct carbon-containing functional groups, corresponding to C-C/C=C (284.8 eV), C-N/C=N (286.2 eV), and O=C-O (288.3 eV) bonds. The spectrum showed the nitrogen signals of C-N=C at 399.8 eV and N-C_3_ at 400.4 eV (Figure 1H). Then, the oxygen signals of the O 1s spectrum revealed the existence of C=O at 531.4 eV and C-O at 532.7 eV (Figure 1I). These results indicated that the *Epimedium*-derived CDs were primarily composed of C, N, and O elements and possessed surface functional groups including -OH, -COOH, -C=O, and -C-O-, which was consistent with the previous results of FTIR spectroscopy. These functional groups not only form the structural basis for their hydrophilicity and excellent dispersibility but are also widely recognized as key sites for their nanozyme catalytic activity [22,23]. By participating in electron transfer and acceptance, they directly promote the SOD-like and CAT-like activities observed in subsequent experiments.

The surface charge characteristics of the CDs were evaluated by measuring their zeta potential. As shown in Figure S2, the zeta potential of the CDs was determined to be −28.07 mV. Previous studies have demonstrated that nanoparticles with zeta potentials exceeding ±20 to ±30 mV exhibited enhanced uptake efficiency across plant cell and chloroplast membranes. Therefore, due to the higher absolute value of the zeta potential, *Epimedium*-derived CDs would be effectively taken up by plant cells.

### 2.2. Antioxidant Multienzymatic Activities and Radical Scavenging Abilities of CDs

**Figure 2:**
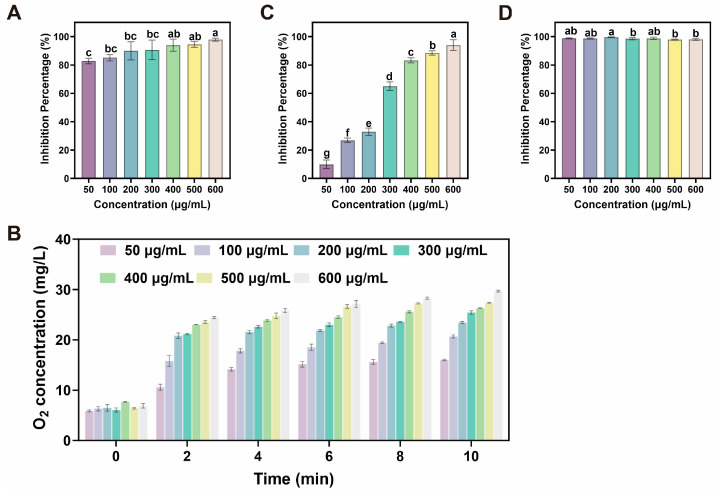
(**A**) Inhibition percentage of O_2_^•−^ at different concentrations of CDs. (**B**) The amount of O_2_ by CDs with different concentrations. (**C**,**D**) Inhibition percentages of ·OH and ABTS^+^·at different concentrations of CDs. Different letters indicate a statistically significant difference by ANOVA (*p* < 0.05).

The antioxidant multienzymatic activities of CDs were firstly investigated. The SOD-mimetic activity of CDs was evaluated by the nitro-blue tetrazolium (NBT) photoreduction method. In this assay, superoxide anion (O_2_^•−^) was generated through riboflavin-mediated photoreduction under aerobic conditions. The resulting O_2_^•−^ reduced NBT to monoformazan, a compound exhibiting a characteristic absorption peak at 560 nm. The O_2_^•−^ scavenging capacity was assessed by monitoring the decrease in absorbance at 560 nm. As illustrated in Figure 2A, the scavenging efficiency increased in a dose-dependent manner with CDs concentration, reaching 93.93 ± 3.45% at 400 μg/mL. After demonstrating the excellent SOD-like activity, the CAT-mimicking activity of the CDs was subsequently investigated. To assess the CAT-mimicking activity of the CDs, their O_2_ generation capacity was evaluated. The introduction of CDs into a H_2_O_2_ solution induced immediate bubble formation, visually demonstrating the rapid catalytic decomposition of H_2_O_2_ into O_2_. This catalytic efficiency was concentration-dependent, as O_2_ production progressively increased with higher CDs nanozyme concentrations (Figure 2B). These findings confirmed that the CDs nanozyme possessed robust CAT-like activity, effectively scavenging H_2_O_2_ to mitigate ROS accumulation and alleviate oxidative stress.

Antioxidant activity analysis is a widely employed approach for evaluating the capacity of materials to neutralize or scavenge free radicals. In this study, the in vitro antioxidant properties of CDs were systematically evaluated using hydroxyl radicals (·OH) and ABTS radical cations (ABTS^+^·) as representative model systems (Figure 2C,D). The results indicated that CDs demonstrated a significant capacity for radical scavenging. This activity displayed a clear concentration-dependent trend. At a concentration of 400 μg/mL, the scavenging percentages for ·OH and ABTS^+^· reached 83.25 ± 1.49% and 98.82 ± 0.68%, respectively. Both values exceeded 80%, indicating strong free radical neutralizing potential. These results confirmed that the as-prepared CDs possessed excellent and concentration-dependent antioxidant activity. We conducted a comparative analysis against a classical antioxidant, vitamin C (VC). As illustrated in Figure S3 at an equivalent concentration of 400 μg/mL, CDs demonstrated superior radical scavenging efficiency, achieving 90.6% for O2•− and 98.55% for ABTS^+^·. These results were notably higher than the 83.17% and 87.82% scavenging rates observed for VC, respectively. We attribute this exceptional activity to the unique chemical composition inherited from the Epimedium precursor, which is rich in intrinsic antioxidant flavonoids and phenolic acids.

Based on these findings, the CDs demonstrated remarkable scavenging capabilities against multiple ROS. Furthermore, based on the observed SOD-like and CAT-like activities, the CDs were demonstrated as a cascade nanozyme capable of sequentially eliminating multiple ROS. The SOD-mimetic activity catalyzed the dismutation of O_2_^•−^ into H_2_O_2_, which was subsequently decomposed into O_2_ and water through the CAT-like activity. This ROS scavenging mechanism mimicked the natural SOD-CAT antioxidant system in plants, thereby establishing a solid foundation for alleviating the oxidative burst triggered by drought stress in vivo.

### 2.3. Effect of CDs on the Growth of Ginseng Seedlings Under Drought Stress

**Figure 3:**
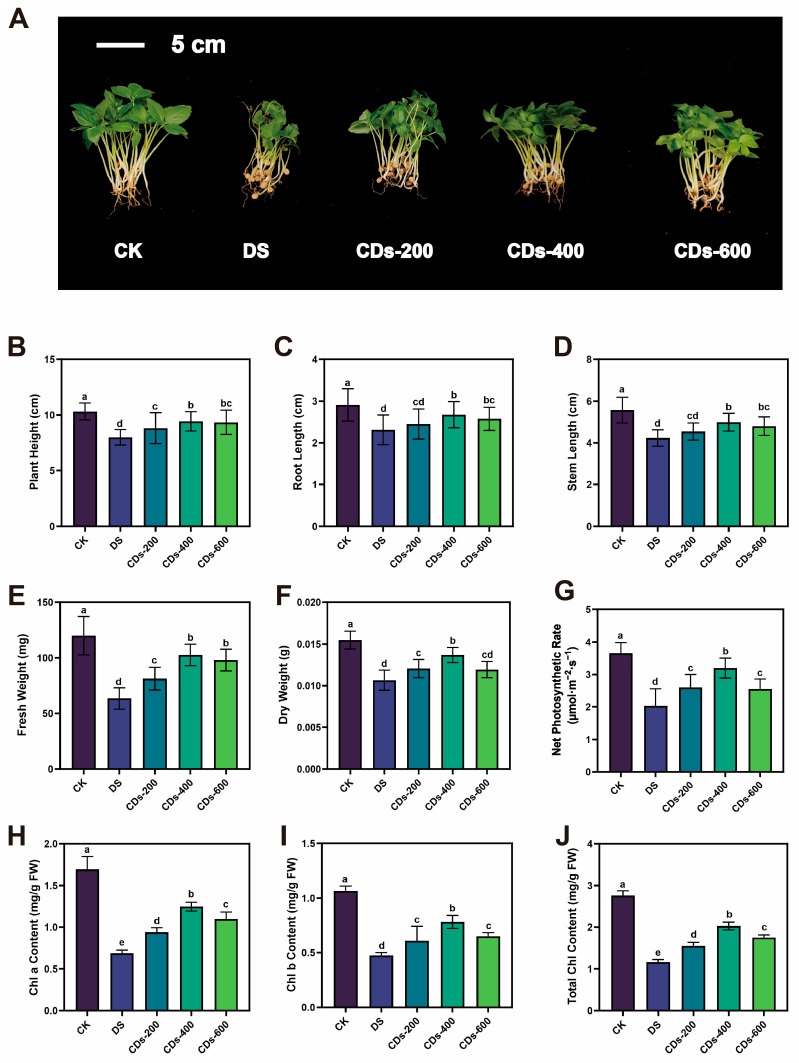
(**A**) Digital photos of CK, DS, and CD treatments on ginseng growth. Ginseng growth was assessed under different treatments: control (CK), drought stress (DS), and CDs at concentrations of 200, 400, and 600 μg/mL (CDs-200, CDs-400 and CDs-600). Effects of CK, DS, and CD treatments on ginseng seedlings for (**B**) plant height, (**C**) root length, (**D**) stem length, (**E**) fresh weight, (**F**) dry weight, (**G**) net photosynthetic rate, (**H**) chlorophyll a content, (**I**) chlorophyll b content, and (**J**) total chlorophyll content. Different letters indicate a statistically significant difference by ANOVA (*p* < 0.05).

Encouraged by the aforementioned in vitro experimental results, ginseng seedlings were employed as a plant model to evaluate the drought stress resistance effect of CDs. Three concentrations of CDs (200, 400, and 600 μg/mL) were applied via foliar spraying to evaluate their antioxidant activity under drought stress conditions. The resulting seedlings were categorized into five experimental groups: control (CK, 70–75% soil moisture), drought-stressed group (DS, 30–36% soil moisture), and three drought-stressed groups supplemented with CDs at concentrations of 200, 400, and 600 μg/mL (CDs-200, CDs-400 and CDs-600, 30–36% soil moisture). The efficacy of this foliar application strategy was critically supported by the outstanding wetting capability of the CDs solution on ginseng leaves. As evidenced in Figure S4, the CDs solution displayed a significantly lower initial contact angle than deionized water, which further decreased over time to 77.2° within one minute. The result indicated that CDs had a good affinity with ginseng leaves, which enabled better diffusion of the active ingredients on the leaves surface and improved the utilization efficiency of CDs. As shown in Figure 3A–F, the biomass exhibited significant dose-dependent enhancement with CD treatment. Increasing the concentration of CDs progressively enhanced the growth parameters, with the most significant enhancement observed at 400 μg/mL. Compared to the drought stress (DS) treatment group, the optimal concentration of CDs elicited significant increases in plant height, root length, stem length, fresh weight, and dry weight by 17.94%, 15.80%, 17.97%, 61.73%, and 28.33% respectively. Similarly, the net photosynthetic rate and chlorophyll content of ginseng seedlings exhibited comparable trends. As illustrated in Figure 3G–J and Figure S5, the net photosynthetic rate, chlorophyll a, chlorophyll b, total chlorophyll, and carotenoid content in the 400 μg/mL CD treatment group increased by 57.75%, 81.06%, 65.00%, 74.51%, and 52.02%, respectively, compared to the DS group. Collectively, these findings validated that the exogenous application of CDs significantly mitigated drought stress-induced growth suppression in ginseng seedlings.

**Figure 4:**
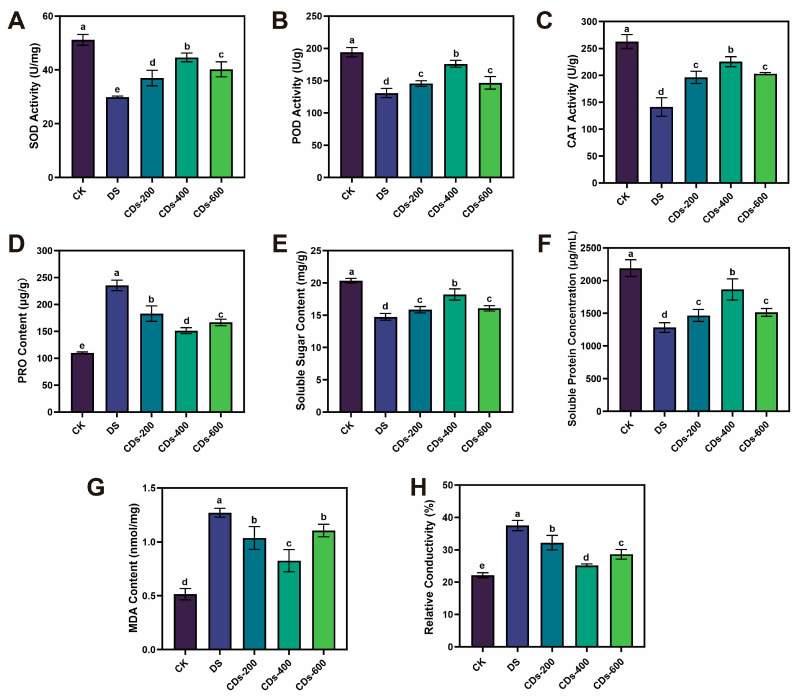
Effect of different concentration of CDs on (**A**) SOD, (**B**) POD, (**C**) CAT activities, (**D**) PRO, (**E**) soluble sugar, (**F**) soluble protein, (**G**) MDA content, and (**H**) relative conductivity. Different letters indicate a statistically significant difference by ANOVA (*p* < 0.05).

Under normal physiological conditions, plants sustain redox homeostasis through balanced generation and scavenging of ROS. Antioxidants, particularly antioxidant enzymes like SOD, POD, and CAT, play a crucial role in protecting plants from oxidative stress by maintaining ROS homeostasis. These enzymes effectively safeguard plants against oxidative damage triggered by excessive ROS production resulting from drought stress [24]. In this study, compared with the DS group, ginseng seedlings treated with CDs exhibited a dose-dependent regulation of antioxidant enzyme activity (Figure 4A–C). The activities of SOD, POD, and CAT enzymes were significantly enhanced at the concentration of 400 μg/mL, reaching 1.49-, 1.35-, and 1.52-fold of the levels in the DS group, respectively. This effect may result from CDs directly scavenging a portion of ROS, thereby alleviating initial oxidative stress on cells. Additionally, CDs may indirectly activate the antioxidant defense system, collectively enhancing the overall antioxidant capacity of cells.

Additionally, plants accumulate various osmolytes to prevent cellular damage under drought stress. Proline (PRO) content, soluble sugar content, and soluble protein content are key indicators of osmotic regulation in plants under stress conditions. As shown in Figure 4D–F, at a concentration of 400 μg/mL CDs, the PRO, soluble sugar, and soluble protein contents were 35.70%, 23.47%, and 45.42% higher than those in the DS group, respectively. Moreover, under drought conditions, elevated levels of ROS in plant cells promote lipid peroxidation, leading to increased malondialdehyde (MDA) production. As depicted in Figure 4G, exogenous application of 400 μg/mL CDs resulted in a 34.95% reduction in MDA content relative to the DS group. Furthermore, relative conductivity is a widely recognized indicator for evaluating cell membrane damage in plant tissues [25]. It quantitatively reflects membrane integrity and stability, with higher relative conductivity indicating more severe membrane disruption. Drought stress induced a significant increase in conductivity (37.53%), which decreased to 25.2% following foliar application of CDs. This reduction indicated that the CD treatment mitigated oxidative damage (Figure 4H). These observations substantiated the efficacy of CDs in mitigating oxidative damage caused by drought stress in ginseng seedlings.

### 2.4. The Role of CDs in Enhanced ROS Scavenging of Ginseng Seedlings Under Drought Stress

**Figure 5:**
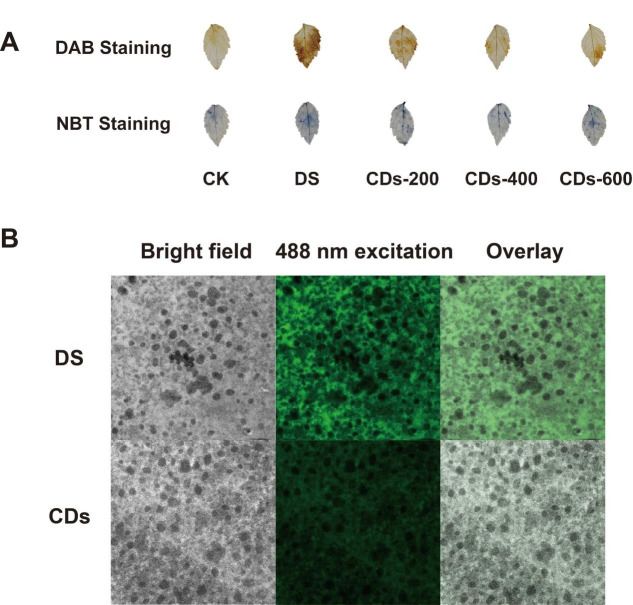
(**A**) DAB and NBT staining for the detection of H_2_O_2_ and O_2_^•−^ in ginseng leaves. (**B**) Level of ROS in mesophyll cells of ginseng leaves were monitored by confocal imaging of DCFH-DA. Scale bar: 20 µm.

To visually confirm the ROS-scavenging efficacy of CDs in vivo, we monitored the accumulation of ROS in drought-stressed ginseng leaves using histochemical staining and fluorescent probes. Firstly, the accumulation of ROS in drought-stressed ginseng leaves was visualized using histochemical staining with 3,3′-diaminobenzidine (DAB) and nitroblue tetrazolium (NBT). The DAB staining enabled visualization of H_2_O_2_ accumulation via the formation of brown precipitates resulting from peroxidase-mediated oxidation reactions. Similarly, NBT staining detected the O_2_^•−^ through its reduction to an insoluble blue formazan product, visually indicating O_2_^•−^ levels in leaf tissues. As shown in Figure 5A, leaves treated with CDs exhibited markedly reduced staining compared to DS group. This was evidenced by much lighter yellowish-brown DAB deposits (indicating H_2_O_2_) and blue NBT formazan deposits (indicating O_2_^•−^), with minimal chromogenic intensity observed at the 400 μg/mL concentration. These results indicated that CD treatment reduced the accumulation of H_2_O_2_ and O_2_^•−^ in ginseng leaves under drought stress.

Additionally, we used 2′,7′-Dichlorodihydrofluorescein diacetate (DCFH-DA) to stain ginseng leaves and employed laser confocal microscopy to detect the distribution of corresponding fluorescence within ginseng cells (with active oxygen in cells presented as green fluorescence). The results of the laser confocal microscopy observations were shown in Figure 5B. Under drought stress, ROS levels were elevated, manifesting as increased DCF fluorescence intensity. CD-treated ginseng cells presented a lower DCF fluorescence signal intensity. Therefore, the CDs promptly removed excessive ROS in drought-stressed ginseng seedlings for reducing cell damage and increasing drought tolerance.

### 2.5. Biosafety Assessment

**Figure 6:**
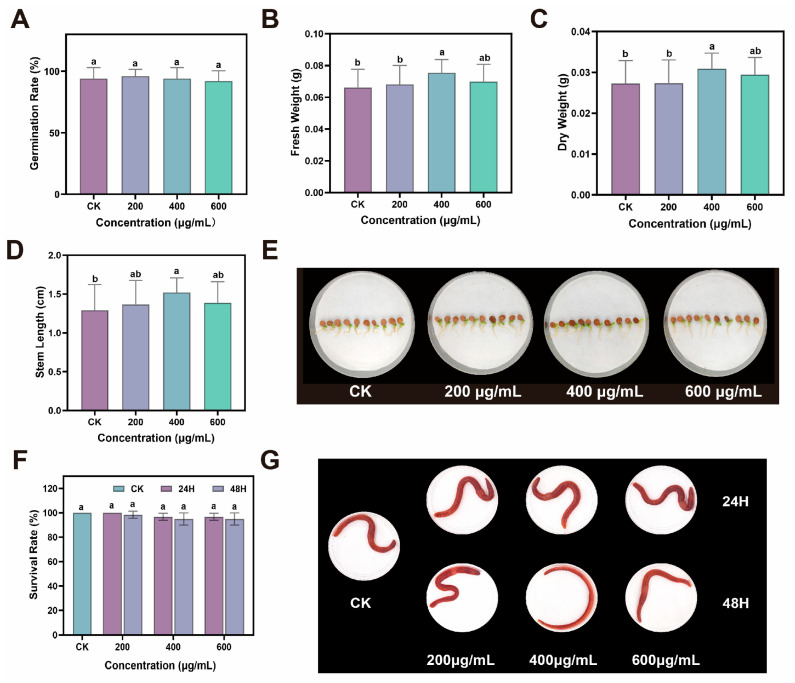
(**A**) Gemination rate, (**B**) fresh weight, (**C**) dry weight, (**D**) stem length, and (**E**) digital photos of ginseng seeds in pure water and treated with CDs. (**F**) Survival rate and (**G**) digital photos of earthworm in pure water and treated with CDs. Different letters indicate a statistically significant difference by ANOVA (*p* < 0.05).

To assess the biocompatibility and environmental safety of CDs, we systematically examined their cytotoxicity to human cells, effects on ginseng seeds growth and acute toxicity to earthworms. Initially, HaCaT cell proliferation and cytotoxicity assays were performed to assess the biosafety of CDs at the cellular level. For concentrations of CDs up to 600 μg/mL, the cellular viability remained above 85% (Figure S6). Subsequently, to evaluate the effects of CDs on plant germination and growth, we immersed ginseng seeds in aqueous solutions of varying CDs concentrations. After 14 days of cultivation, all treated groups exhibited germination rates exceeding 90% with robust growth (Figure 6A–E). Furthermore, considering that nanomaterials can enter aquatic or terrestrial ecosystems through pathways such as rainfall runoff, posing potential risks to non-target organisms, we also assessed their acute toxicity to earthworms. Earthworms were exposed to CDs at concentrations of 200, 400, and 600 μg/mL. Mortality was recorded after 24 and 48 h of exposure. Results showed that survival rates in all CD-treated groups exceeded 90%, with no significant acute toxicity observed (Figure 6F). In addition, earthworms in all dose groups maintained normal behavioral responses to external tactile stimuli, which indicated no evident neurotoxic or physiological impairment under the tested conditions (Figure 6G). Collectively, these results demonstrated that CDs exhibited minimal biological toxicity and possessed favorable environmental safety.

### 2.6. Mechanisms Analysis for the Mitigation of Oxidative Stress in Ginseng Seedlings by CDs

**Figure 7:**
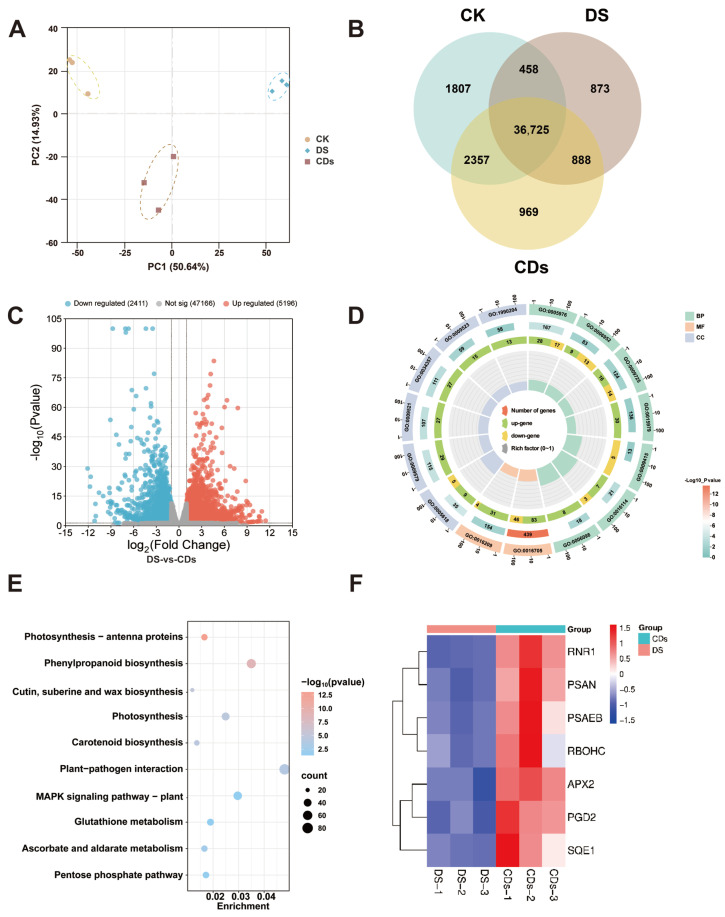
(**A**) PCA of genes from various treatments, including CK, DS and CD groups. (**B**) Venn diagram quantifying shared and unique DEGs among CK, DS and CD groups. (**C**) Volcano plot of expressed genes between DS and CDs group. (**D**) GO enrichment analysis of differentially expressed genes between DS and CDs group. (**E**) KEGG pathway analysis of differentially expressed genes between DS and CDs group. (**F**) Heatmap of differentially expressed genes between different treatments, including DS and CDs.

To elucidate the molecular mechanisms underlying the CD-mediated alleviation of oxidative stress, we performed transcriptomic analysis on three sample groups: healthy control plants (CK group), drought-stressed plants (DS group), and plants treated with CDs under drought stress (CDs group). Principal component analysis (PCA) demonstrated that the transcriptomic profile of the CD-treated group was clearly distinct from those of the other groups (Figure 7A). Based on Venn diagram analysis, 873 and 969 genes were specifically induced in ginseng leaves under DS and CD treatment, respectively (Figure 7B). “|Fold Change| ≥ 1, *p* < 0.05” was chosen as the filtering criteria to analyze the differential expression genes between the two groups. Comparative analysis between the DS and CD groups identified 7607 differentially expressed genes (DEGs), including 5196 upregulated and 2411 downregulated genes (Figure 7C). Collectively, these transcriptomic data provided a foundation for subsequent functional enrichment analysis to decipher the key biological processes regulated by CDs.

Gene Ontology (GO) enrichment analysis and Kyoto Encyclopedia of Genes and Genomes (KEGG) pathway enrichment analysis were performed on the DEGs from the DS vs. CDs comparison. GO analysis revealed that differentially expressed genes constituted hierarchically structured regulatory networks across multiple critical biological pathways. In GO analysis, the synergistic activation of redox enzyme activity (GO:0016209) and photosynthesis (GO:0015979) pathways provided a molecular explanation for the observed increases in net photosynthetic rate and reductions in photooxidative damage. The enhanced carbohydrate metabolism (GO:0005976) pathway not only supported energy supply but also provided the metabolic basis for the observed accumulation of osmotic regulators such as soluble sugars and proline. Notably, the downregulation of the water response pathway (GO:0009415) alongside the upregulation of defense (GO:0006952) and hormone response (GO:0009725) pathways transcriptomically elucidated the adaptive strategy of redirecting resources from growth to defense. This aligned with observed shifts in biomass allocation under drought stress (Figure 7D). KEGG pathway analysis further revealed the precise molecular mechanisms regulated by CD treatment. The enrichment of photosynthesis-antenna protein (ath00196) and photosynthesis (ath00195) pathways directly correlated with increased chlorophyll content and restored net photosynthetic rate. Meanwhile, the activation of glutathione metabolism (ath00480) corroborated enhanced SOD, POD, and CAT enzyme activities, collectively explaining the physiological phenomena of reduced MDA content and decreased electrolyte leakage. The MAPK signaling pathway (ath04016), acting as a central hub, coordinated phenylpropanoid biosynthesis (ath00940) through the plant-pathogen interaction pathway (ath04626). This networked regulatory model elucidated the holistic activation of the antioxidant defense system at the signal transduction level. The enrichment of the carotenoid biosynthesis pathway (ath00906) further enhanced the plant’s photoprotection capacity and antioxidant levels, providing additional support for the observed maintenance of photosynthetic function (Figure 7E).

At the gene expression level, regulatory patterns of key genes established direct mechanistic links with specific physiological improvements. Significant upregulation of photosystem key genes *PSAN* (3.79-fold) and *PSAEB* (2.94-fold) provided the molecular basis for increased chlorophyll content and restored net photosynthetic rate in the CD-treated group. Enhanced expression of the antioxidant gene *APX2* (2.63-fold) corroborated with elevated SOD, POD, and CAT enzyme activities in the experiment, collectively mediating efficient ROS scavenging. This correlation fully explained the physiological phenomena of reduced MDA content and decreased electrolyte leakage. The elevated expression of the signaling hub *RBOHC* (2.85-fold) linked MAPK signaling to antioxidant defense, maintaining ROS homeostasis by coordinating antioxidant enzyme expression. The upregulation of the metabolic *PGD2* (2.01-fold) bridged energy metabolism with the glutathione antioxidant system. The NADPH it supplied simultaneously supported the detoxification function of *RNR1* (3.38-fold), explaining the systemic enhancement of antioxidant capacity from an energy supply perspective. Notably, the 8-fold upregulation of *SQE1*, a gene involved in phenylpropanoid defense compound synthesis, pinpointed a critical mechanism through which CD treatment enhanced physical and chemical barriers by regulating secondary metabolism. These coordinated gene expression changes collectively constructed a multi-level regulatory network spanning signal perception, energy metabolism, ROS scavenging, and defense synthesis, systematically elucidating the intrinsic mechanism by which CD treatment enhanced plant drought resistance at the physiological level (Figure 7F).

## 3. Discussion

Due to their excellent physicochemical properties and high biocompatibility, biomass-derived CDs have become highly regarded green and environmentally friendly nanomaterial for boosting plant stress resistance. This study successfully synthesised multifunctional CDs from the medicinal plant Epimedium using a simple one-step hydrothermal method. The as-prepared CDs exhibited uniform dispersion, a small particle size of approximately 6.8 nm, and abundant surface functional groups (such as -OH, -COOH, and -C=O), collectively conferring hydrophilicity. Most studies indicated that biomass-derived CDs primarily consist of C, N, and O elements [26]. Similarly, FT-IR and XPS analyses identified these three principal elements within the Epimedium-derived CDs. Notably, these CDs exhibited potent cascade nanozyme activity via simultaneously mimicking the characteristics of SOD and CAT for effectively scavenging multiple ROS in a dose-dependent manner. These in vitro antioxidant properties provided a robust foundation for their application in enhancing drought tolerance in ginseng seedlings.

Drought stress severely restricts plant growth by disrupting cellular equilibrium and inducing oxidative damage. Our research demonstrated that foliar application of CDs significantly mitigates drought-induced growth inhibition in ginseng, evidenced by increases in plant height, root length, and biomass, alongside improved photosynthetic performance. Notably, at the optimized concentration of 400 μg/mL, CDs restored net photosynthetic rates and chlorophyll content to levels approaching those of healthy plants, indicating a substantial recovery of photosynthetic function under stress conditions. These findings aligned with prior research demonstrating that CDs enhanced plant photosynthetic capacity and increasing biomass in adverse environments [27]. Furthermore, CDs significantly reduced the accumulation of ROS and the content of MDA, while concurrently enhancing the activities of key antioxidant enzymes and osmoprotectants. This synergistic enhancement of antioxidant defense and osmotic adjustment underscores the crucial role of CDs in maintaining redox homeostasis and protecting membrane integrity under drought conditions. Similar effects have been documented for CDs derived from *Salvia miltiorrhiza*, which were reported not only to scavenge ROS directly but also to mobilize Ca^2+^ signaling, thereby enhancing plant adaptation under abiotic stress [28]. These findings suggested that ROS scavenging represents a common mechanism through which biomass-derived CDs alleviate abiotic stress. Moreover, the regulation of ROS homeostasis is critically dependent on an efficient antioxidant defense system. The enhanced activities of SOD, CAT, and POD, which are essential for ROS scavenging under drought stress, were strongly activated by CDs in drought-stressed ginseng seedlings. Consistent with our observations, the induction of similar antioxidant activities by functional CDs has been reported, which improved plant tolerance under drought conditions through stimulating antioxidant enzymes, modulating osmotic balance, and concomitantly enhancing photosynthetic performance [29].

Transcriptome analysis further elucidated the molecular mechanisms underlying CD-induced drought tolerance. Differential gene expression analysis highlighted key alterations in pathways to photosynthesis (e.g., *PSAEB*), antioxidant defence (e.g., *APX2*), MAPK cascade signalling (e.g., *RBOHC*), and phenylpropanoid biosynthesis (e.g., *SQE1*), collectively illustrating a multi-level regulatory network activated by CDs. The observed upregulation of the MAPK cascade is of particular significance, as this highly conserved signalling system is a central regulator of plant responses to diverse biotic and abiotic stresses, including drought-induced osmotic stress and ROS signalling [30]. Within this network, the increased expression of photosynthesis- and antioxidant-related genes, coupled with the coordinated modulation of the MAPK pathway and the downregulation of water-responsive pathways, suggested that CDs facilitate a strategic reallocation of cellular resources from primary growth to defence activation under stress conditions. Furthermore, cytotoxicity assays, seed germination tests, and earthworm toxicity studies confirmed the biosafety and environmental friendliness of *Epimedium*-derived CDs. This outcome aligned with the biosafety profiles of other biomass-derived CDs [31]. Their high safety profile makes them well-suited for use as environmentally friendly agents in agriculture. From a broader perspective, this study not only expands the application of biomass-derived CDs in medicinal plants but also highlights their function as ROS scavengers.

Although we confirmed through integrated physiological, biochemical, and transcriptomic analyses that *Epimedium*-derived CDs alleviate drought stress by scavenging reactive oxygen species (ROS) and activating complex defense signaling networks, we acknowledge that this study failed to directly visualize the distribution and localization of CDs within ginseng tissues. Nevertheless, confocal microscopy using the DCFH-DA probe directly confirmed significantly reduced ROS levels within mesophyll cells treated with carbon dots (Figure 5B), corroborating the ROS-scavenging activity of carbon dots within plants. This phenomenon aligns with the documented behavior of similarly sized surface-charged carbon dots, which can efficiently penetrate leaf surfaces via stomata and be internalized by plant cells [32]. Transcriptomic data revealing marked physiological improvements and synergistic transcriptional reprogramming of antioxidant and stress response pathways strongly supports the proposed mechanistic model. Future studies will employ fluorescently labeled CDs or high-resolution microscopy techniques to precisely track the uptake, transport, and localization of these nanoparticles within ginseng plants.

Our findings demonstrated that *Epimedium*-derived CDs enhanced drought tolerance in ginseng through a coordinated mechanism. This process involved both the direct scavenging of multiple ROS and the activation of the antioxidant enzyme system, which collectively functioned to maintain cellular homeostasis. These insights not only corroborated but also significantly extended previous studies on plant-derived CDs, underscoring the immense potential of *Epimedium*-derived CDs as a green and environmentally friendly nanomaterial for boosting plant resilience and productivity in drought-stressed environments. Our one-pot hydrothermal process is inherently green, utilizing only deionized water and avoiding toxic solvents or reagents, which significantly lowers its environmental footprint compared to conventional nanomaterial synthesis. Most importantly, the exceptional multi-enzyme mimetic activity of the *Epimedium*-derived CDs enables them to function effectively at very low concentrations (e.g., 400 μg/mL). This high potency means that a minimal quantity of material is required to treat a large agricultural area. While this research confirmed the effectiveness of *Epimedium*-derived CDs in improving drought tolerance, several aspects deserve further attention. Future studies should examine the long-term effects of CDs on soil health and microbial communities, track their movement and accumulation in plants over time, and explore possible synergistic effects with other agents. In addition, large-scale field trials will be essential to verify the practical use and sustainability of CDs in real farming environments, facilitating their adoption in climate-resilient agriculture.

## 4. Materials and Methods

### 4.1. Materials

3,3′-diaminobenzidine (DAB) and nitroblue tetrazolium (NBT) were purchased from Macklin Biochemical Technology Co., Ltd. (Shanghai, China). Hydrogen peroxide (H_2_O_2_) was obtained from Aladdin Biochemical Technology Co., Ltd. (Shanghai, China). All biochemical assay kits were purchased from Nanjing Jiancheng Bioengineering Institute (Nanjing, China). Human epidermal keratinocytes (HaCaT) (cell line FH0186) were sourced from Shanghai Fuheng Biotechnology Co., Ltd. (Shanghai, China).

### 4.2. Synthesis of CDs

*Epimedium* was utilized as a carbon source for the synthesis of CDs. Specifically, the raw Epimedium leaves were dried in an oven at 60 °C for 24 h to a constant weight. The dried leaves were subsequently pulverized into a fine powder using a commercial grinder and sieved through a 100-mesh sieve to ensure uniformity. Then, 0.1 g of the powder was dispersed in 4 mL of water. The mixture was sealed in a Teflon-lined stainless-steel autoclave and subjected to hydrothermal treatment at 180 °C for 8 h. As the autoclave was cooled to the room temperature, the crude product was dissolved into 4 mL of ultrapure water. Next, the previous solution was centrifuged for 15 min at 8000 rpm to achieve the removal of the undissolved particles, and the supernatant was therewith collected. Finally, the obtained solution was filtered with a 0.22 µm of filter membrane and further purified through the dialysis process (membrane of 1000 MWCO) for 12 h, then stored at 4 °C before use. The yield of the CDs was calculated to be approximately 13.7%, based on the weight of the initial leaf powder and the final collected CDs product.

### 4.3. Characterization

The morphological characteristics of the specimens were examined using a Tecnai G2 F20 transmission electron microscope (FEI Company, Hillsboro, OR, USA). Quantitative assessment of particle size distribution was performed through statistical analysis of TEM images utilizing ImageJ software (Version 1.8). Chemical functionalization of the nanoparticles was characterized by Fourier transform infrared spectroscopy (FTIR) employing a Thermo Scientific Nicolet iS20 spectrometer (Thermo Fisher Scientific, Madison, WI, USA). Optical absorption properties were investigated using a Shimadzu UV-1900i ultraviolet-visible spectrophotometer (Shimadzu Corporation, Kyoto, Japan). Surface elemental composition and chemical states were determined through X-ray photoelectron spectroscopy (XPS) measurements conducted on a Thermo Fisher Scientific K-Alpha spectrometer (Thermo Fisher Scientific, Waltham, MA, USA).

### 4.4. SOD Mimetic Assays

The SOD-like catalytic activity of CDs was assessed based on the inhibition of nitro-blue tetrazolium (NBT) photoreduction. A reaction mixture was prepared in 400 μL of phosphate-buffered saline (PBS, 10 mM, pH 7.4) containing riboflavin (8 μL, 1 mM), NBT (3 μL, 10 mM), L-methionine (26 μL, 0.2 mM), and varying amounts of CDs. This mixture was exposed to UV light for a predetermined duration, after which its absorbance at 560 nm was recorded. A control sample, consisting of the same reaction mixture, was kept in the dark throughout the procedure. All absorbance measurements were performed using a SHIMADZU UV-1900i spectrophotometer. The inhibition rate was calculated according to the following Equation:

(1) $$Inhibition\;Rate\;\left(\%\right)=\left(1-\frac{A1-A2}{A0}\right)\times 100$$

where *A*0 is the absorbance of the control without CDs, *A*1 is the absorbance in the presence of CDs, and *A*2 is the absorbance of CDs.

### 4.5. CAT Mimetic Assays

The CAT-like enzymatic activity of CDs was evaluated through the detection of dissolved oxygen. The assay was performed in 50 mL centrifuge tubes, each containing 35 mL of PBS (0.01 M, pH 7.4) and 1 mL of CDs solution at various concentrations. Thereafter, 4 mL of 30% H_2_O_2_ solution was introduced into each tube under continuous stirring. Variations in oxygen content within the reaction system were monitored over time using a portable dissolved oxygen meter, with measurements recorded at 2-min intervals for a total of five time points.

### 4.6. Hydroxyl Radical Scavenging Activity of CDs

The hydroxyl radical (·OH) scavenging activity was assayed by the Fenton reaction. First, ·OH was generated based on the reaction of FeSO_4_ (9 mM) and H_2_O_2_ (8.8 mM). After 10 min, SA (9 mM) and various concentrations of CDs were added to the above mixture. After 30 min, the ·OH scavenging percentage was determined by measuring the absorbance of 2,3-dihydroxybenzoic acid at 510 nm, which was generated by the reaction of SA with ·OH. The calculation method is as follows:

(2) $$Inhibition\;Rate\;\left(\%\right)=\left(1-\frac{A1-A2}{A0}\right)\times 100$$

where *A*0 is the absorbance of the control without CDs, *A*1 is the absorbance in the presence of CDs, and *A*2 is the absorbance of CDs.

### 4.7. ABTS Radical Scavenging Activity of CDs

The ABTS radical scavenging activity was determined according to the following method. An ABTS^+^·cation radical stock solution was generated by reacting ABTS (7.4 mM) with K_2_S_2_O_8_ (2.6 mM). This stock solution was then diluted to a specified concentration to obtain the working solution. Subsequently, CDs at varying concentrations were added to the ABTS working solution. After the mixture was incubated in darkness for 5 min, the absorbance of the resulting solution was measured at 734 nm. The scavenging rate was calculated using the following formula:

(3) $$Inhibition\;Rate\;\left(\%\right)=\left(1-\frac{A1-A2}{A0}\right)\times 100$$

where *A*0 is the absorbance of the control without CDs, *A*1 is the absorbance in the presence of CDs, and *A*2 is the absorbance of CDs.

### 4.8. Plant Growth and Cultivation

Surface-sterilized ginseng seeds were thoroughly rinsed with distilled water. Thirty seeds were sown in each pot (14 cm diameter × 17.2 cm height) filled with soil, and a total of ten pots were set up per treatment group. The resulting seedlings were categorized into five experimental groups: control (CK, 70–75% soil moisture), drought-stressed group (DS, 30–36% soil moisture), and three drought-stressed groups supplemented with CDs at concentrations of 200, 400, and 600 μg/mL. (CDs-200, CDs-400 and CDs-600, 30–36% soil moisture). Following 40 days of cultivation under these conditions, the seedlings were harvested. Various physiological and biochemical indicators were measured.

### 4.9. Determination of Net Photosynthetic Rate

The net photosynthetic rate of ginseng leaves was determined using LCpro-SD photosynthesizer at 9–11 am in sunny weather with temperature (16–20 °C). Multiple repetitions of the measurements were averaged. Each group underwent ten replicate measurements.

### 4.10. Measurement of Chlorophyll Content

Different groups of 0.2 g of fresh ginseng leaves were weighed and cryogenically ground. Ethanol was added to each sample, and the homogenate was ground until it turned white, then allowed to stand for 5 min. The homogenate was filtered, concentrated to 25 mL, and the absorbance of the solution was measured at 665 nm, 649 nm, and 470 nm, respectively.

### 4.11. Determination of Biochemical and Physiological Indexes

The plant height, root length, stem length, fresh weight and dry weight were measured. In addition, the activities of superoxide dismutase (SOD), catalase (CAT), peroxidase (POD), proline (PRO), malondialdehyde (MDA), soluble sugar content, and soluble protein content were measured by corresponding reagent kits.

### 4.12. Determination of Relative Conductivity of Leaves

A 0.5 g sample was accurately weighed, cut into segments, and transferred into a stoppered tube. After adding 10 mL of distilled water, the mixture was agitated on an orbital shaker for 30 min and then allowed to stand for an additional 30 min. The initial conductivity (*C*1) was measured using a SANXIN MP521 conductivity meter (Shanghai Sanxin Peirui Instrument Technology Co., Ltd., Shanghai, China). The sample was then heated in a boiling water bath for 30 min, cooled to room temperature, and the final conductivity (*C*2) was measured. The relative conductivity was calculated according to the following formula:

(4) $$Relative\;Conductivity\;\left(\%\right)=\left(C1/C2\right)\times 100$$

where *C*1 is the initial conductivity, *C*2 is the final conductivity.

### 4.13. DAB and NBT Staining

A solution of either 3,3′-diaminobenzidine (DAB) and nitroblue tetrazolium (NBT) was prepared by dissolving 50 mg of the respective powder in 100 mL of distilled water, with complete dissolution facilitated by ultrasonication. Leaves from ginseng seedlings were immersed in the staining solution (10 mL) within a six-well plate. The staining process was carried out in the dark under constant agitation at 80–100 rpm. Following an incubation period of 12–16 h, the samples were subjected to decolorization using absolute ethanol in a 95 °C water bath. The ethanol was replaced repeatedly until complete removal of chlorophyll, as indicated by the disappearance of green pigmentation. The decolorized specimens were subsequently mounted and photographed for analysis.

### 4.14. Intracellular ROS Scavenging Detection

CDs powder was initially dissolved in TES infiltration buffer, with the buffer alone serving as the control. Each solution was slowly injected into separate plant leaves. Following a 3-h incubation period, leaf disks were excised from the infiltrated leaves using a cork borer and subsequently treated with 25 μM H_2_DCF-DA in the dark. After 30 min of incubation, the leaf disks were rinsed three times with distilled water and gently blotted dry. The prepared samples were mounted on glass slides and carefully sealed with an anti-fade mounting medium to prevent interference from air bubbles. Fluorescence imaging was finally performed using a confocal laser scanning microscope, with signal acquisition conducted under 488 nm laser excitation.

### 4.15. Biocompatibility and Environmental Safety Evaluation

HaCat cells at the logarithmic growth phase were plated in 96-well plates and maintained in DMEM containing 10% fetal bovine serum and 1% penicillin/streptomycin at 37 °C for 24 h. The medium was then replaced with fresh medium incorporating varying concentrations of CDs, followed by another 24 h of incubation. Cell viability was determined by the MTT assay.

Seed germination phytotoxicity assay. CDs were diluted to three concentrations (200, 400, and 600 μg/mL). Ten milliliters of each solution was added to Petri dishes (Φ = 90 mm) lined with two filter papers. Ten surface-sterilized ginseng seeds were placed in each dish. Plates were incubated in a growth chamber at 25 °C for 14 days, with ultrapure water serving as the control (five replicates per treatment). Germination rates and growth conditions were recorded.

Acute toxicity test of earthworms (filter paper contact test). Prepare multiple porous and breathable earthworm preservation boxes, lay a filter paper in each box, drop different concentrations of CDs aqueous solution (200, 400, 600 μg/mL) onto the filter paper to fully wet it. Then place the earthworm in it. Each box held 20 earthworms, with three replicates per group. After it evaporates, replenish the fluid promptly. All earthworm preservation boxes should be stored in a dark environment at 20 ± 1 °C and 80–85% relative humidity for 24 and 48 h. Record the mortality rate of earthworms and take photos at 24 and 48 h. If earthworms do not respond to mild mechanical stimuli, they are considered dead.

### 4.16. RNA Sequencing and Transcriptome Analysis

Fresh ginseng leaves were used for transcriptome sequencing, and three biological replicates were taken for each treatment. Total RNA was isolated from plant tissues using the TIANGEN RNAprep Pure Plant Plus Kit (DP441, TIANGEN Biotech Co., Ltd., Beijing, China) specifically designed for polysaccharide and polyphenol-rich samples. Approximately 100 mg of tissue was pulverized in liquid nitrogen and vigorously homogenized in Lysis Buffer SL containing β-mercaptoethanol. The homogenate was clarified by centrifugation, and the resultant supernatant was filtered, mixed with absolute ethanol, and applied to the CR3 silica-membrane column. The bound RNA underwent a comprehensive on-column DNase I digestion to remove genomic DNA, followed by sequential washes with Buffer RW1 and Buffer RW. Pure, integral total RNA was finally eluted in RNase-free water. RNA quality was assessed by confirming A260/A280 ratios between 1.8–2.1 and visualizing intact 28S and 18S rRNA bands on a denaturing agarose gel.

The RNA sequencing library was constructed using total RNA as the initial input material. Poly(A) RNA was selectively isolated through hybridization to oligo(dT)-conjugated magnetic beads. The enriched mRNA was subsequently subjected to fragmentation via incubation with divalent cations under elevated temperature in 5× First Strand Synthesis Reaction Buffer. First-strand cDNA synthesis was carried out using random hexamer primers and M-MuLV Reverse Transcriptase (RNase H^−^). Second-strand cDNA was then generated through the coordinated action of DNA Polymerase I and RNase H. The resulting double-stranded cDNA fragments were blunt-ended by exonuclease/polymerase treatment, followed by 3′ end adenylation. Adapters featuring a hairpin loop structure were ligated to the prepared fragments. Library fragments within the preferred size range of 370–420 bp were selectively purified using the AMPure XP system (Beckman Coulter, Brea, CA, USA). Amplification was performed with Phusion High-Fidelity DNA Polymerase employing Universal PCR primers and Index (X) Primer. The final PCR products were purified using the AMPure XP system, and library quality was verified using the Agilent Bioanalyzer 2100 system (Agilent Technologies, Santa Clara, CA, USA). Finally, index-coded samples were clustered on a cBot Cluster Generation system (Illumina, San Diego, CA, USA) with the TruSeq PE Cluster Kit v3-cBot-HS (Illumina, San Diego, CA, USA) following the protocols. After cluster generation, the library preparations were sequenced on an Illumina Novaseq platform (Illumina, San Diego, CA, USA) and 150 bp paired-end reads were generated.

### 4.17. Statistical Analysis

Statistical analyses were performed using SPSS Statistics (Version 27.0.1) software. Data are presented as mean ± standard deviation (SD). All treatments included at least three independent replicates. Differences were considered statistically significant at *p* < 0.05.

## 5. Conclusions

In this study, multifunctional antioxidant CDs were successfully synthesized from the medicinal herb *Epimedium* via a simple one-step hydrothermal method. The CDs exhibited excellent dispersion, small particle size, and abundant surface functional groups. In vitro antioxidant experiments demonstrated that the CDs possessed remarkable cascade nanozyme activities mimicking both SOD and CAT, along with potent free radical scavenging ability. Under drought stress, foliar application of CDs significantly alleviated the inhibition of ginseng seedling growth by drought through regulating the antioxidant enzyme system, improving osmotic balance, and reducing membrane lipid peroxidation. Transcriptomic analysis further revealed that CDs activated key pathways related to photosynthesis, antioxidant defense, and stress signaling, thereby reconstructing a regulatory network. Moreover, safety assessments confirmed the excellent biocompatibility and environmental safety of the CDs. Overall, this work presents a novel, green, and eco-friendly strategy for enhancing drought tolerance in medicinal plants, offering promising prospects for application in environmentally friendly agriculture.

## Data Availability

Data are contained within the article or Supplementary Materials.

## Supplementary Materials

The following supporting information can be downloaded at https://www.mdpi.com/article/10.3390/plants14233705/s1. Figure S1: Fluorescence intensity of CDs solution stored for different times. Figure S2: The Zeta potential of CDs. Figure S3: (A) O_2_^•−^ and (B) ABTS^+^· radical inhibition percentage at different concentrations. Figure S4: Contact angle on the surface of ginseng leaves. Figure S5: Effect of different treatments on ginseng seedlings for carotenoid content. Figure S6: Cytotoxicity assessment of different concentrations of CDs.
