# Supplementary material for: Epimedium-Derived Multi-Antioxidant Carbon Dots Nanozymes for Mitigating Drought Stress of Ginseng Seedlings
Source: Plants (Basel). 2025 Dec 4;14(23):3705. doi: 10.3390/plants14233705 (PMC12693751; doi:10.3390/plants14233705)
Supplement: Supplementary file 1 [file plants-14-03705-s001.zip › plants-3996671-supplementary.pdf]

Supplementary Materials

Epimedium-derived multi-antioxidant carbon dots nanozymes for mitigating drought stress of ginseng seedlings

Yanghong Liu [1], Tong Wu [1], Jialong He [1], Chunyao Shang [1], Jiaheng Li [1], Yu Dong [1], Huiyuan Xie [1], Chen Xu [1,2*], Yingping Wang [1,2*] and Kai Dong [1,2*]

1 College of Chinese Medicinal Materials, Jilin Agricultural University, Changchun 130118, China

2 National & Local Joint Engineering Research Center for Ginseng Breeding and Development, Changchun 130118, China

*Corresponding Author: Chen Xu (E-mail: xuchen@jlau.edu.cn), Yingping Wang (wangyingping@jlau.edu.cn) and Kai Dong (dongkai@jlau.edu.cn).

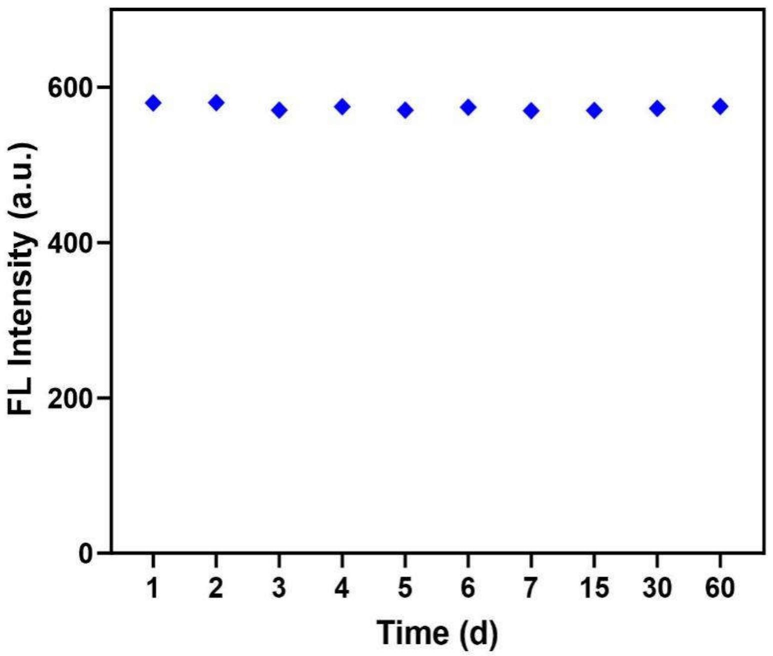

Figure S1. Fluorescence intensity of CDs solution stored for different times.

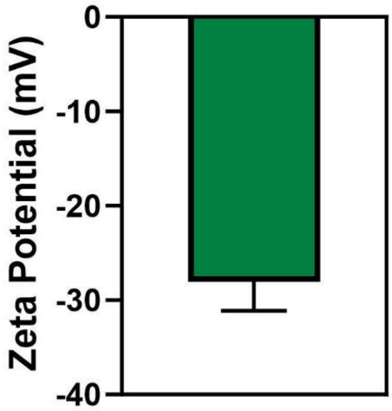

Figure S2. The Zeta potential of CDs.

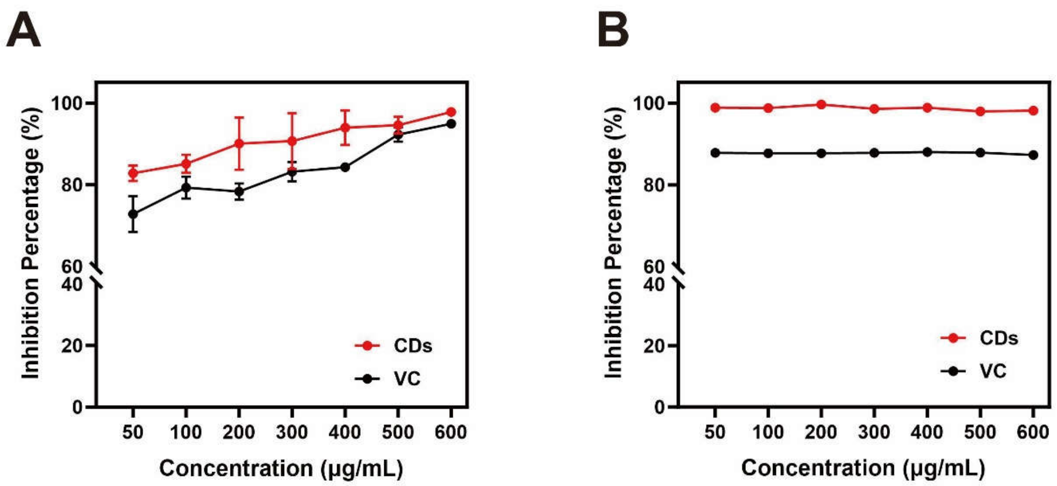

Figure S3. (A) $O_2{\cdot}^-$ and (B) $ABTS^{+\cdot}$ radical inhibition percentage at different concentrations.

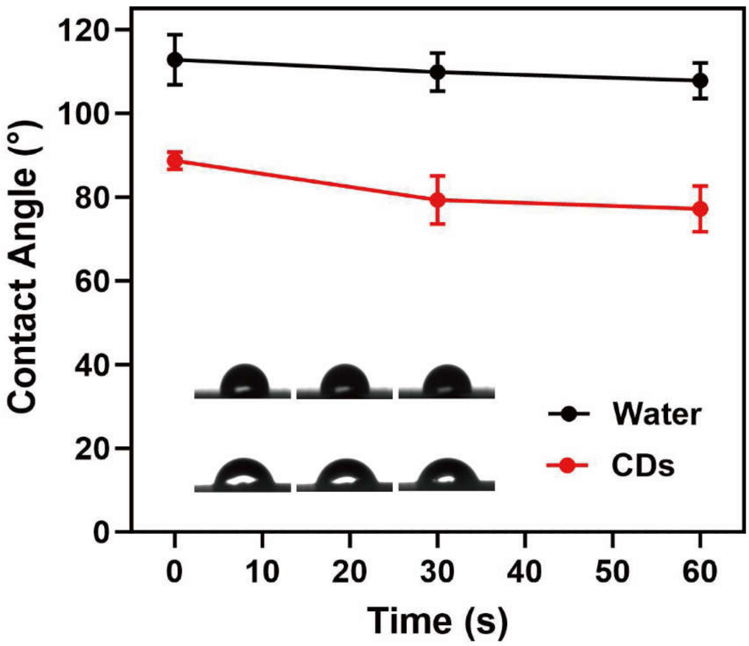

Figure S4. Contact angle on the surface of ginseng leaves.

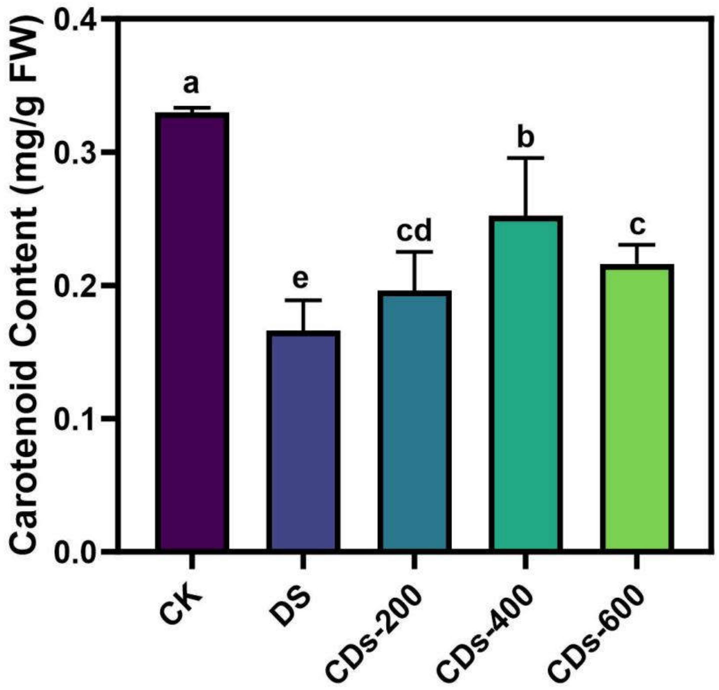

Figure S5. Effect of different treatments on ginseng seedlings for carotenoid content.

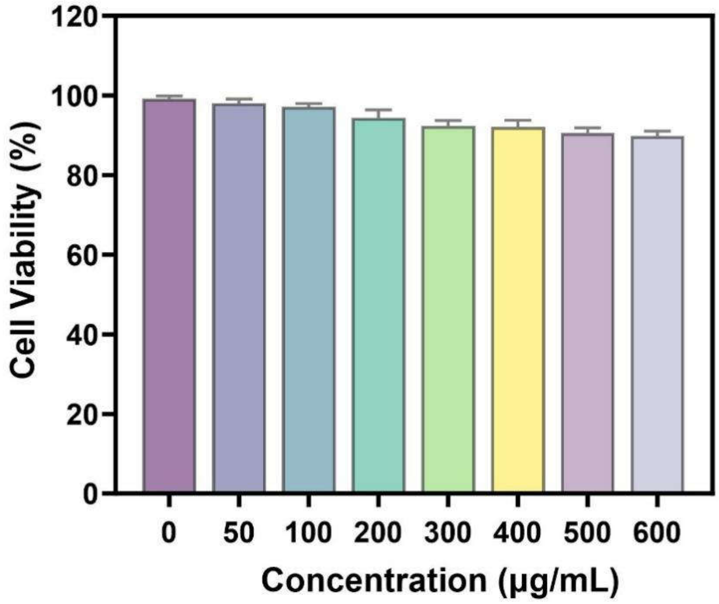

Figure S6. Cytotoxicity assessment of different concentrations of CDs.
